# Efficacy and safety of cytokines versus first-line sunitinib and second-line axitinib for patients with metastatic renal cell carcinoma (ESCAPE study): A study protocol for phase III randomized sequential open-label study

**DOI:** 10.1016/j.conctc.2019.100403

**Published:** 2019-06-26

**Authors:** Yoshifumi Kadono, Hiroyuki Konaka, Kouji Izumi, Satoshi Anai, Kiyohide Fujimoto, Kei Ishibashi, Noriyasu Kawai, Taku Kato, Akinori Iba, Naoya Masumori, Kenichi Yoshimura, Atsushiu Mizokami

**Affiliations:** a Department of Integrative Cancer Therapy and Urology, Kanazawa University Graduate School of Medical Science, Kanazawa, Japan; b Department of Urology, Nara Medical University, Kashihara-Shi, Nara, Japan; c Department of Urology, Fukushima Medical University, Fukushima, Japan; d Department of Nephro-urology, Nagoya City University Graduate School of Medical Sciences, Nagoya, Japan; e Department of Urology, Gifu University Graduate School of Medicine, Gifu, Japan; f Department of Urology, Wakayama Medical University, Wakayama, Japan; g Department of Urology, Sapporo Medical University School of Medicine, Sapporo, Japan; h Innovative Clinical Research Center (iCREK), Kanazawa University Hospital, Kanazawa, Japan

**Keywords:** Cytokine, Interferon alfa, Interleukin-2, Sunitinib, Axitinib, Metastatic renal cell carcinoma

## Abstract

Appropriate protocol for the sequential treatment of metastatic renal cell carcinoma (mRCC) has not been established yet. Some mRCC cases with favorable risk were reported to achieve complete remission and durable response using interferon alfa (IFNα) + low dose interleukin-2 (IL-2). Cytokine therapies may be suitable for some patients with mRCC as first-line therapy. The present study is a phase III, investigator-initiated, multicenter, prospective randomized controlled trial investigating patients with low and intermediate risk mRCC classified by Memorial Sloan-Kettering Cancer Center risk criteria to evaluate the efficacy and safety of sequential treatment with cytokine (IFNα + IL-2) as first-line and axitinib as second-line therapy versus sequential treatment with sunitinib as first-line and axitinib as second-line therapy, which is the current standard treatment for patients with favorable risk. The target sample size was set at 72 patients per group (total 144 cases). The study duration is 7 years, and the duration for recruitment is 4 years. Our expectation of this trial is to clarify first- and second-line sequential treatment for mRCC better, especially in patients with favorable risk and some with intermediate risk. The results of this trial will certainly contribute to new information for the strategy of first- and second-line sequential treatment for mRCC.

**Trial registration:**

University hospital Medical Information Network (UMIN) Center identifier UMIN 000012522.

## 1 Introduction

Recently developed molecular target drugs and immune checkpoint inhibitors have changed treatment strategies for metastatic renal cell carcinoma (mRCC) because of their efficacy; however, complete recovery from mRCC remains very rare [[1], [2], [3]]. Therefore, our current realistic goal for mRCC treatment would be maximum elongation of overall survival (OS) of patients while maintaining their quality of life (QOL). To achieve the goal, we must plan longitudinal treatment strategies that consider the general condition of the patient and control adverse events (AEs) of administered drugs. However, an appropriate protocol for sequential treatment of mRCC has yet to be established. The number of patients who can undergo treatment is gradually decreasing, as the treatment lines are increasing to second-, third-, and fourth-line, because the general condition of patients worsens with disease progression and/or AEs of the drugs. To extend OS of patients with mRCC as long as possible, selecting the first-line and consecutive second-line therapy is extremely important. Based on the guidelines for mRCC treatment and the current Japanese system of national insurance, the recommended drug selection for mRCC categorized as favorable risk by the Memorial Sloan-Kettering Cancer Center (MSKCC) criteria [4,5] is sunitinib [1] or pazopanib [3] as first-line drug, and axitinib [2] or nivolumab [6] as second-line. However, OS in patients treated with cytokines, which have been used for a long time for mRCC in Japan, has been reported to be better than in western countries [7]. Therefore, it is speculated that the effectiveness of cytokines is better for Japanese patients than for western patients. Some mRCC cases meeting favorable risk criteria were reported to have achieved complete remission (CR) and durable response using interferon alfa (IFNα) + low dose interleukin-2 (IL-2) [8,9]; however, molecular targeted treatments may not achieve CR for patients with mRCC. Cytokine therapies are not effective in patients with high risk mRCC, but cytokine therapy may be effective for some patients with intermediate risk mRCC, because patients with intermediate risk mRCC are part of a heterogenous group [10].

We planned a prospective randomized controlled trial (RCT) for patients with low and intermediate risk mRCC (classified by MSKCC risk criteria) to evaluate the efficacy and safety of sequential treatment of cytokine (IFNα + IL-2) as first-line and axitinib as second-line therapy versus sequential treatment of sunitinib as first-line and axitinib as second-line therapy, which is the current standard treatment for favorable risk mRCC (ESCAPE Study).

## 2 Material and methods

### 2.1 Aim of the study

To assess the efficacy and safety of cytokines versus sunitinib as first-line therapy followed by axitinib as second-line therapy in the treatment of patients with mRCC.

### 2.2 Study design

**Fig. 1:**
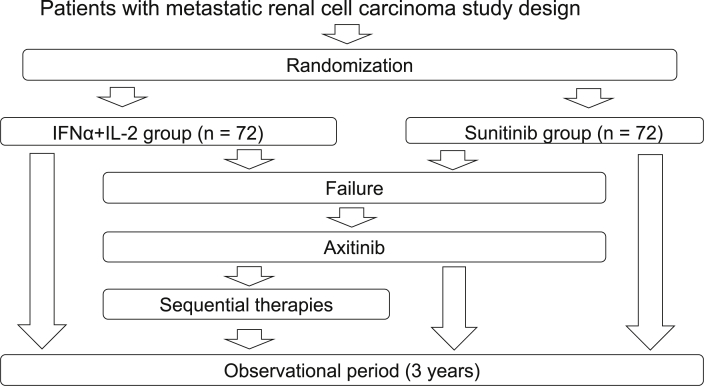
Trial registration: UMIN000012522.

The present study is a phase III, investigator-initiated, multicenter RCT involving a head-to-head comparison of IL-2 plus IFNα vs. sunitinib as first-line therapy followed by axitinib as second-line therapy for patients with mRCC. Patients will be randomly assigned to IL-2 plus IFNα or sunitinib-axitinib treatment group, as shown in Fig. 1.

### 2.3 Additional measures

A validated health-related QOL (HRQOL) questionnaire, the European Organization for Research and Treatment of Cancer (EORTC) QLQ-C30, the Functional Assessment Of Cancer Therapy-Kidney Symptom Index-Disease Related Symptoms (FKSI-DRS), and the EuroQol Group (EQ)-5D that has been translated into Japanese will be administered before treatment, at 2 and 4 months after the beginning of first-line treatment, and at the time of completion or drop out of second-line treatment to comprehensively evaluate the various aspects of physical and psychosocial well-being.

### 2.4 Eligibility criteria: inclusion criteria

Patients are eligible to participate in the study if they:

1. Have nephrectomy with mRCC;
2. Have clear cell RCC diagnosis confirmed;
3. Have had no prior systemic treatment for mRCC;
4. Have MSKCC risk criteria of favorable or intermediate;
5. Have at least one measurable lesion on computed tomography (CT) or magnetic resonance imaging (MRI) at the baseline per the Response Evaluation Criteria in Solid Tumors (RECIST) 1.1 criteria;
6. Are aged 20–80 years;
7. Have Eastern Cooperative Oncology Group (ECOG) performance status of 0 or 1;
8. Have acceptable hematopoietic function defined as neutrophil ≥1,500/mm^3^, platelet ≥10 times 10^4^/mm^3^, hemoglobin >9.0 g/dL;
9. Have acceptable hepatic function defined as total bilirubin ≤1.5 times ULN, AST and ALT ≤2.5 times ULN (patients with hepatic metastasis ≤5.0 times ULN);
10. Have acceptable renal function defined as serum creatinine ≤2.0 times ULN;
11. Have a life expectancy of more than 3 months; and
12. Have provided informed consent for participation in the study after they received explanation of the briefing document.

### 2.5 Ineligibility criteria: exclusion criteria

Patients are ineligible to participate in the study if they:

1. Have a history of hypersensitivity against IFNα, IL-2, sunitinib, or axitinib;
2. Are currently pregnant or suspected to be pregnant or are nursing, or plan to have a baby (including men);
3. Have a history of hypersensitivity to biological preparations such as vaccines;
4. Use Shou-Sai-Kotou (special herbal drug);
5. Have autoimmune hepatitis;
6. Have a history of interstitial pneumonia;
7. Have been treated for another primary malignancy within 3 years of enrollment; and
8. Fulfill investigator judgment of ineligibility.

### 2.6 Informed consent: ethics approval

This study was conducted in accordance with the Declaration of Helsinki revised in 2008 and Ethical Guidelines for Clinical Research revised in 2008. All the treatment and examinations for mRCC are provided after written informed consent is provided. The institutional ethics committees of the participating institutions approved the ESCAPE study for mRCC.

### 2.7 Methods of recruitment and random allocation

Recruitment was from October 2013 to September 2017. Eligible patients were randomly assigned to one of two treatment groups through the data center at the Innovative Clinical Research Center Kanazawa University (iCREK). Randomization was centrally performed by Waritsukekun (Mebix, Tokyo, Japan) using stratified sampling method to obtain adequate between-group balance for age (<70 or ≥ 70), MSKCC risk (favorable or intermediate), status of metastasis (lung only or other than lung), and participating institution.

### 2.8 Administration of IFNα plus IL-2 vs. first-line therapy with sunitinib followed by second-line with axitinib

**Fig. 2:**
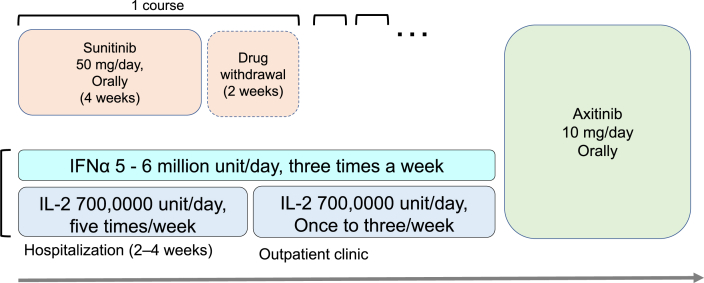
Administration schedule.

Patients assigned to cytokine group receive intravenous injection of IL-2 at a dose of 700,000 unit/day five days/week for the initial 2 or 4 weeks, and then, one or three times/week as maintenance plus subcutaneous injection of IFNα 5–6 million units two or three times/week (Fig. 2). Sunitinib at a dose of 50 mg/day is orally administered to patients who are assigned to the sunitinib group, and one course consists of 4 weeks of administration and 2 weeks of withdrawal. A schedule of 2 weeks of administration and one week of withdrawal is also permitted. Reduction of doses is permitted if a clinical investigator considers the basic doses inappropriate for any reason. The administration of IL-2 plus IFNα or sunitinib is terminated when progressive disease (PD) is confirmed based on RECIST v.1.1, the patient dies, or severe AEs occur. A clinical investigator administers axitinib as second-line treatment immediately after confirmed comprehensive evaluation of PD based on RECIST v.1.1 during first-line treatment. In case of cessation of first-line treatment because of severe AEs, a clinical investigator may consider restarting first-line treatment after recovery below grade 2. Axitinib at a dose of 10 mg/day (5 mg dose twice per day) is orally administered to patients as second-line treatment. Dose reduction or dose increase up to 20 mg/day (10 mg dose twice per day) is permitted if a clinical investigator considers the basic doses inappropriate for any reason. The administration of axitinib is terminated when PD is confirmed by comprehensive evaluation based on RECIST v.1.1, the patient dies, or severe AEs corresponding to discontinuation criteria occur. Zoledronic acid and denosumab are permitted for patients with bone metastasis.

### 2.9 Data collection

**Fig. 3:**
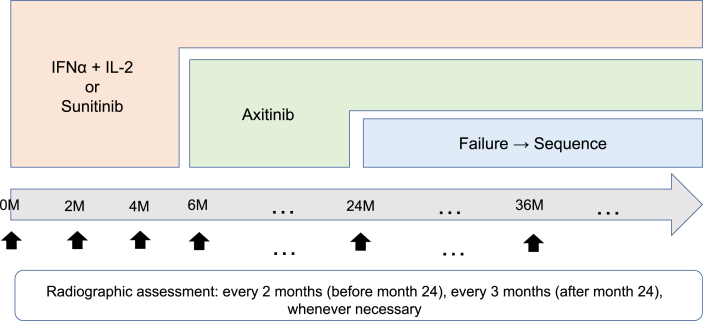
Follow-up schedule.

All the patients provide written informed consent to participate in the study and are asked to complete a medical history. Clinical data obtained in the ESCAPE study include the ECOG performance status, physical examination findings (e.g., height, body weight, body temperature, and blood pressure), results of hematologic examination (e.g., red blood cell count, hemoglobin, hematocrit, platelet count, white blood cell count, and differential count of leucocytes), results of blood biochemical examination (e.g., liver enzymes, alkaline phosphatase, total bilirubin, total protein, albumin, creatinine, lipids, fasting blood sugar, hemoglobin A1c, C-reactive protein, and electrolytes), results of urinalysis, chest X-ray imaging, lung to pelvic CT or MRI, brain CT or MRI, bone scintigraphy, electrocardiography, and the questionnaires for HRQOL (e.g., EORTC QLQ-C30, FKSI-DRS, EQ-5D). The X-ray, brain CT, and bone scintigraphy are performed at the time of study registration. Other examinations are performed every 2 months from the date of the commencement of treatment to month 24 and every 3 months after month 24 until the study is completed (Fig. 3). However, examinations can be performed at any time, if the clinical investigator considers them to be necessary.

### 2.10 Definition of endpoints

The primary endpoint is total progression-free survival (PFS) from randomization to PD based on RECIST v.1.1 criteria examined with CT or MRI or death during second-line therapy. PD during first-line therapy is not counted as an event based on the definition. In case of cancellation during first-line therapy caused by severe AEs or refusal of patient without confirming PD, the end date of first-line therapy is not counted as cessation, and the case continues to receive the second-line therapy.

### 2.11 Seven secondary endpoints are set in the ESCAPE study

1. OS, defined as the time from randomization to death from any cause.
2. Each PFS in first-line treatment and second-line treatment based on RECIST v.1.1 criteria.
3. Objective response rate (ORR), defined as the proportion of CR and partial response (PR) cases in total cases based on best overall response using RECIST v.1.1 criteria, and disease control rate (DCR), defined as the proportion of CR, PR, and stable disease (SD) cases in total cases in first-line and second-line treatment.
4. Time to treatment failure (TTF), defined as the time from commencement of first-line therapy to earliest time of PD using RECIST v.1.1 criteria, death from any cause, or cancellation of therapy in first-line treatment.
5. Total TTF from first-line to second-line treatment, defined as the time from commencement of first-line therapy to earliest time of PD using RECIST v.1.1 criteria, death from any cause, or cancellation of the therapy in second-line treatment.
6. Safety in first-line and second-line treatment evaluated by Common Terminology Criteria for Adverse Events v4.0 system.
7. HRQOL in first-line and second-line treatment evaluated by EORTC QTQ-C30, FKSI-DRS, and EQ-5D.

### 2.12 One exploratory endpoint is set in the ESCAPE study

1. Applied medicine, PFS, ORR, and DCR in third-line and fourth-line treatment.

### 2.13 Sample size consideration and anticipated completion of enrollment

Based on the AXIS trial (second-line axitinib versus sorafenib) reported by Rini et al. [2] and the reports from Akaza et al. [11], the median total TTF would be calculated as 26.1 (10.4 + 15.7) months for combination of INFα + IL-2 and axitinib, and 15.3 months (10.5 + 4.8) for the combination of sunitinib and axitinib. In this study, hypothetical median TTFs were set for 26 months in the combination of INFα + IL-2 and axitinib and 15 months for the combination of sunitinib and axitinib. We calculated the sample size from the study duration of 5 years (two-year entry period and three-year observation period) and the difference in total TTF between the groups. To detect a significant difference between the groups by a log-rank test with significant level of 0.05 and power of 80%, at least 65 patients in each group are required. Furthermore, given the assumption that approximately 10% of randomized patients will not be evaluated for various reasons, the target sample size was set at 72 patients per group (total 144 cases).

The final patients were enrolled in September 2017, and the entire study will be completed by September 2020.

### 2.14 Planned statistical analyses

Intention-to-treat analyses in each group will be performed, and survival curves will be estimated with Kaplan-Meier method. A log-rank test will be used to evaluate differences in the survival curves between the two groups of patients. Hazard ratio will be estimated with the Cox proportional hazard model. Restricted mean survival time will be considered to quantify the treatment benefit in the absence of proportional hazard assumption in survival curves. ORR (CR + PR) and DCR (CR + PR + SD) in each group using the best response evaluated by RECIST v1.1 will be estimated for 95% confidence interval with Clopper-Pearson method. AEs in each group will be aggregated by grades and types, and the proportion of AEs will be compared between the groups by Fisher's exact test. The chronological changes in the HRQOL from the baseline evaluation using EORTC QLQ-C30, FKSI-DRS, and EQ-5D will be compared. All the tests will be two-sided, and a *P*-value of 0.05 will be considered statistically significant.

## 3 Discussion

According to recent guidelines for mRCC developed as a result of large RCTs, for favorable risk classified by MSKCC criteria [5] or the International mRCC Database Consortium criteria [4], a sequential treatment of sunitinib as first-line therapy [1] and nivolumab as second-line therapy [6] is recommended. For intermediate and high risk cases, a sequential treatment of ipilimumab + nivolumab as first-line therapy [12] and vascular endothelial growth factor (VEGF)-targeted drugs [2] is recommended with high evidence level.

Sunitinib is recommended for first-line treatment for favorable risk mRCC. It is a multitarget oral tyrosine-kinase inhibitor (TKI) that selectively blocks signal transduction from receptor tyrosine kinase (RTK), contributing to tumor cell proliferation, tumor angiogenesis, and metastasis. Sunitinib blocks activities of RTK such as platelet-derived growth factor receptor (PDGFR)-alfa and -beta; vascular endothelial growth factor receptor (VEGFR)-1, −2, and −3; mast/stem cell growth factor receptor (c-kit); and FMS-like tyrosine kinase 3 [13,14]. Motzer et al. reported the efficacy of sunitinib as first-line treatment for mRCC compared with conventional treatment. An RCT between IFNα and sunitinib for 750 mRCC cases revealed 11 months PFS in the sunitinib group compared with 5 months PFS in the IFNα group (hazard ratio 0.42, P < 0.001), and better ORR in sunitinib group (31%) than that in the IFNα group (6%) (P < 0.001) [1].

Axitinib is one of the recommended second-line treatment for mRCC, and it selectively and strongly blocks the activities from VEGFR-1, VEGFR-2, and VEGFR-3. These regulate angiogenesis and lymphangiogenesis as the main regulator, and in addition to the efficacy for mRCC, it is anticipated that the selectivity for VEGFR will reduce the toxicity caused by other TKIs that target multiple RTK [15]. AXIS trial, which is an RCT evaluating the efficacy of second-line treatment for 715 cases of mRCC using sorafenib or axitinib, reported better median PFS in the axitinib group (6.8 months) than in the sorafenib group (4.7 months) (hazard ratio 0.665, P < 0.001). There was no significant difference in the proportion of treatment cessation because of toxicity between axitinib (4%) and sorafenib (8%) [2].

Reported response rate (RR: CR + PR) of IFNα or IL-2 monotherapy was about 10%–20% [16]. United States Food and Drug Administration approved IL-2 as high dose IL-2 (600,000–700,000 IU/kg x 5 days in a two-week cycle); however, the dose of 70,000–210,000 IU/kg/day IL-2 approved in Japan was low [17,18]. Koreth et al. reported in 2011 that the administration of one million IU/day induced IL-2 regulatory T cell (Treg) for 2–3 months and controlled the occurrence of graft versus host disease (GVHD) [19]. The usual administration dose of 1.05 million IU/day for IL-2 would contribute to reinforcement of activity of natural killer cells (NK) and lymphokine activated killer cells (LAK). However, it could induce Treg and possibly reduce the antitumor effect such as NK or LAK activity. It has been reported that IFNα suppresses Treg [20]; therefore, the combination therapy of low dose IFNα and IL-2 would contribute to antitumor activity without reduction caused by Treg. Akaza et al. reported that the combination therapy of IFNα and low dose IL-2 for 42 cases of mRCC resulted in approximately 35% ORR, two cases of CR, approximately 70% DCR, 10.4 months PFS, and approximately 70% 3-year OS. These results were favorable compared with IFNα or IL-2 monotherapy [11]. The main AEs with combination therapy were fever and general malaise, which were controllable using medicines such as antipyretics. Ito et al. reported that Japanese patients with mRCC could live longer than patients in western countries undergoing IFNα therapy. Using the genetic analysis of Japanese patients, patients were able to achieve long-term survival with a good response of IFNα against mRCC [21]. Based on these reports, cytokine therapy may contribute to long-term survival for some patients with mRCC, especially Japanese patients. In fact, Naito et al. reported excellent clinical outcomes for patient with mRCC in Japan during the cytokine treatment era compared with the outcomes from the reports in western countries [7,22]. Chow et al. reported that IL-2 treatment for mRCC showed 48.1% ORR and 21.6% CR among selected patients with favorable pathology based on constitution of histological growth pattern [23]. Cytokine therapies have the potential to achieve complete and durable response for a limited group of patients with mRCC; therefore, cytokine therapy might be a selectable option as first-line treatment especially for Japanese patients with mRCC with favorable risk criteria.

Each ORR of axitinib as second-line after sunitinib as first-line and after cytokine as first-line treatment was 11.3% and 32.5%, and each PFS was 4.8 months and 12 months in the AXIS trial, respectively. Therefore, axitinib was more effective after cytokine than after sunitinib [2]. Escudier et al. reported that each ORR of sorafenib 800 mg as second-line after disease progression using cytokine as first-line and sorafenib 1200 mg as second-line after disease progression using sorafenib 800 mg as first-line treatment was 20% and 5.2%, respectively. Namely, sorafenib was more effective after cytokine than dose escalation [24]. Moroto et al. reported that each excellent ORR and DCR of sorafenib after 6- or 12-week administration of combination of IFNα and IL-2 was 44.4% and 94.4%, respectively [25].

In some cases of mRCC, especially in Japanese patients whose response to cytokine therapy would be good, the duration of response by the first-line cytokine and second-line TKI could be longer than that of first-line TKI treatment and second-line treatment with another agent. Conversely, AEs of sunitinib in Japanese patients tend to be more severe than in Westerners; therefore, management for the AEs of sunitinib is sometimes difficult, even though we had accumulated experience of AEs with sunitinib. To improve the outcome of sequential treatment for mRCC, we planned a prospective randomized controlled open-label trial between first-line IFNα + IL-2 and second-line axitinib and first-line sunitinib and second-line axitinib, which is considered as a standard first- and second-line treatment for patients with mRCC with favorable risk. Our expectation of this trial is it will clarify better first- and second-line sequential treatment for mRCC, especially in patients with favorable risk and some patients with intermediate risk. In doing so, the results of the trial will definitely contribute to new information for the strategy of first- and second-line sequential treatment for patients with mRCC.

## Funding

ESCAPE study has not received any external funding.

## Availability of data and materials

The data supporting the conclusions of this article will not be available until the final report of this trial to avoid bias on the analysis.

## Ethics approval and consent to participate

ESCAPE study received approval from Medical Ethics Committee of Kanazawa University first (reference number: 2013–023), and subsequently from the institutional ethics committees of all other participating 9 hospitals listed below; Kanazawa University; Medical Ethics Committee of Kanazawa University; Nara Medical University; Medical Ethics Committee of Nara Medical University, Fukushima Medical University; Ethics review committee of Fukushima Medical University, Nagoya City University Graduate School of Medical Sciences; Nagoya City University Graduate School of Medical Sciences Ethics Committee, Gifu University; institutional Ethics Committee of Gifu University Hospital, Wakayama Medical University; Ethical Committee of Wakayama Medical University, Sapporo Medical University School of Medicine; ethical committee of Sapporo Medical University, Nagasaki University Hospital; Medical Ethics Committee of Nagasaki University Hospital; ethics committee of Tokyo Medical and Dental University, Dokkyo Medical University; Ethics Committee of Dokkyo Medical University Koshigaya Hospital.

## Conflicts of interest

None declared.
